# Transcutaneous electrical stimulation at auricular acupoints innervated by auricular branch of vagus nerve pairing tone for tinnitus: study protocol for a randomized controlled clinical trial

**DOI:** 10.1186/s13063-015-0630-4

**Published:** 2015-03-19

**Authors:** Tian-Tian Li, Zhao-Jun Wang, Song-Bai Yang, Jun-Hong Zhu, Shi-Zhong Zhang, San-Jin Cai, Wen-Han Ma, Ding-Qi Zhang, Zhi-Gang Mei

**Affiliations:** Medical College of China Three Gorges University, No. 8, University Avenue, Yichang, Hubei China; Yichang Hospital of Traditional Chinese Medicine, Clinical Medical College of Traditional Chinese Medicine, China Three Gorges University, Yichang, Hubei 443003 China

**Keywords:** auricular acupoints, auricular branch of vagus nerve (ABVN), randomized controlled trial, subjective tinnitus, tones

## Abstract

**Background:**

Subjective tinnitus is a phantom sensation experienced in the absence of any source of sound. Its mechanism remains unclear, and no approved drugs are available. Vagus nerve stimulation (VNS) is an exciting new method to treat tinnitus, but direct electrical stimulation of the cervical vagus has disadvantages. This randomized controlled clinical trial aims to overcome these limitations by stimulating the auricular branch of vagus nerve (ABVN) on the outer ear. Since the ABVN is the only peripheral branch of the vagus nerve distributed on the ear’s surface, it should be possible to achieve analogous efficacy to VNS by activating the central vagal pathways. However, researches have indicated that the curative effect lies in a combination of auditory and vagal nerve stimulation. Moreover, from traditional Chinese theory, auricular acupoints used to treat tinnitus are mainly in the regions supplied by the ABVN. Whether stimulation at the auricular acupoints is due to unintentional stimulation of vagal afferent fibers also needs evidence.

**Methods/design:**

A total of 120 subjects with subjective tinnitus are randomized equally into four groups: (1) electrical stimulation at auricular acupoints (CO10, CO11, CO12, and TF4) innervated by the ABVN; (2) electrical stimulation at auricular acupoints (CO10, CO11, CO12, and TF4) innervated by ABVN pairing tones; (3) electrical stimulation at auricular acupoints innervated by non-ABVN pairing tones; (4) electrical acupuncture. Patients will be treated for 30 minutes every other day for 8 weeks. The primary outcome measure is the Tinnitus Handicap Inventory. The secondary outcome measure combines a visual analogue scale to measure tinnitus disturbance and loudness with the Hospital Anxiety and Depression Scale. Assessment is planned at baseline (before treatment) and in the 4th and 8th week, with further follow-up visits after termination of the treatment at the 12th week. Any adverse events will be promptly documented.

**Discussion:**

Completion of this trial will help to confirm whether ABVN or the combination of ABVN and sound stimulus plays a more important role in treating tinnitus. Moreover, the result of this clinical trial will enhance our understanding of specific auricular acupoints.

**Trial registration:**

Chinese Clinical Trials Register ChiCTR-TRC-14004940.

## Background

Subjective tinnitus is considered as a phantom sensation experienced in the absence of any internal or external acoustic stimulus. It can be perceived as a hissing, buzzing, humming, roaring, whistling, or ringing sound in one or both ears, or somewhere in the head. At present, tinnitus has becoming the most common otology problem, affecting 10% to 30% of the general population and an estimated prevalence of 24.2% in the older population [1]. Meanwhile, with the growing popularity of electronic music, young people experience transient or permanent tinnitus at a ratio of 89.5% and 14.8%, respectively, after exposure to loud music [2]. Among severe tinnitus sufferers, tinnitus-related sensations, such as anxiety, annoyance, frustration, and depression lead to a negative impact on quality of life in 70% of those affected [3], and they are usually told that they must learn to live with it. Yet the pathological mechanisms that underlie tinnitus perception remain largely hypothetical and are still not well understood. Currently, the universally accepted hypothesis is that interactions between altered cochlear inputs and distorted central auditory processing provoke tinnitus. Studies have supported that subjective tinnitus may be a result of the expression of neural plasticity and that anomalies may develop because of decreased input from the ear, deprivation and sound stimulation, overstimulation or other factors as yet unknown. For the buried pathological mechanisms, treatment options are limited. No physiotherapy treatments can yet be considered sufficient in providing long-term reduction of tinnitus impact and there is no drug approved by the European Medicines Agency or the Food and Drug Administration on the market [4,5]. Therefore, an effective and safe therapy for tinnitus is of considerable importance to meet this significant unmet clinical need.

Vagus nerve stimulation (VNS) is approved by the Food and Drug Administration for both refractory epilepsy and resistant depression [6,7]. VNS offers an exciting new perspective for the treatment of tinnitus and it has recently been demonstrated that VNS is a promising method [8]. In this method, an electrode surgically implanted around the left cervical vagus nerve is connected to a pulse generator placed subcutaneously in the upper chest. However, considering the disadvantages of the implantation, including lesions of the vagus nerve, infection, shortness of breath, possible mechanical failure of electronic equipment, and battery replacement, a relatively safe and well tolerated improved method is desired. To minimize side effects, stimulation of the auricular branch of vagus nerve (ABVN) has been suggested [9]. The ABVN is the only peripheral branch of the vagus nerve distributed on the surface of ear, and studies using the transganglionic horseradish peroxidase method [10,11] have shown that it mainly projects to the nucleus of the solitary tract in the brainstem. Functional magnetic resonance imaging and vagus sensory evoked potentials revealed that ABVN stimulation shows considerable similarity to implanted VNS, with both acting through the central auditory pathway of the vagus nerve [12,13]. Thus, it would be safer simply to deliver mild electric shocks to the skin of the outer ear instead of resorting to VNS with surgery. However, some researchers reported that ABVN stimulation alone seems to have no relevant improvement of tinnitus complaints [8,14], and it is suggested that the combination with other interventions, such as sound stimuli, which would reduce the cortical response to mid-frequency tones, increase frequency selectivity, and decrease cortical synchronization [15] should be considered. But there is not currently enough evidence in clinical practice of the effect of the method of pairing tones with stimulation at ABVN.

Moreover, auricular acupuncture, the underlying mechanism of which is unclear, has been used to treat tinnitus for thousands of years [16]. Auricular acupoints applied to treat tinnitus such as Kidney (CO10), Yidan (CO11), Liver (CO12) and Shenmen (TF4) are distributed in the regions supplied by ABVN [17]. So whether auricular acupoints are due to an unintentional stimulation of the vagal afferent fibers also needs more clinical and experimental evidence.

Therefore, we have designed a randomized, four-arm, controlled clinical study to provide a conclusive answer. In this trial, our aim is to attempt to confirm whether ABVN or the combination of ABVN and sound stimulus plays a more important role in the treatment of tinnitus. Moreover, the result of this clinical trial will enhance our understanding of specific auricular acupoints.

## Methods/design

### Design

**Figure 1:**
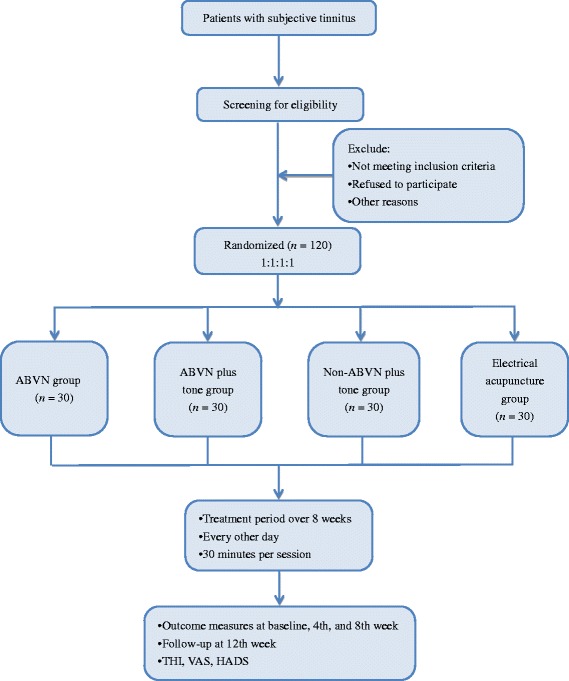
**Flow chart.** ABVN, auricular branch of vagus nerve; HADS, Hospital Anxiety and Depression Scale; THI, Tinnitus Handicap Inventory; VAS, visual analogue scale.

This is a randomized, single-blind, four-armed, controlled clinical trial conducted in Yichang Hospital of Traditional Chinese Medicine, Clinical Medical College of Traditional Chinese Medicine, China Three Gorges University. Study patients will be screened based on specific inclusion and exclusion criteria. Before beginning the treatment, all candidates will undergo a standard neuro-otological examination and a baseline audiometric assessment, including measurement of hearing thresholds, minimal masking levels, loudness discomfort levels, and stapedial reflexes, performed by professional audiologists. Then they will be equally randomized into four groups: (1) electrical stimulation at auricular acupoints (CO10, CO11, CO12, and TF4) innervated by ABVN (the ABVN group); (2) electrical stimulation at auricular acupoints (CO10, CO11, CO12, and TF4) innervated by ABVN pairing tones (the ABVN plus tone group); (3) electrical stimulation at auricular acupoints innervated by non-ABVN pairing tones (the non-ABVN plus tone group); (4) an electrical acupuncture group. Detailed information regarding the clinical procedures is presented in Figure 1. The clinical endpoints are assessed by blinded independent statisticians. Any adverse effects during the course will be reported. This trial was registered on the Chinese Clinical Trial Register (ChiCTR-TRC-14004940).

The trial will be performed according to the principles of the Declaration of Helsinki (Edinburgh version, 2000). In addition, written informed consent will be obtained from all participants. Ethical permission was obtained from the Research Ethical Committee of Yichang Hospital of Traditional Chinese Medicine, Clinical Medical College of Traditional Chinese Medicine, China Three Gorges University. The ethics committee approval number is 201407041.

### Recruitment period and methods

Patients will be enrolled in Yichang Hospital of Traditional Chinese Medicine, Clinical Medical College of Traditional Chinese Medicine, China Three Gorges University with a target sample size of 120 subjects. We will recruit the participants by advertising in the hospitals, in public newspapers and on the internet homepages of hospitals. The trial will be executed from July 2014 to July 2015.

### Types of participant

#### Inclusion criteria

Qualified participants meeting all of the following conditions will be recruited [18,19]:

1. Single-tone subjective tinnitus, typical conditions of unilateral or bilateral;
2. Age 15 to 65 years, either sex;
3. Recurrent attacks for more than 1 month or persist attacks for more than 5 days;
4. Must be able to hear stimulation tones presented by the device at all frequencies;
5. Must be able to complete the forms and use the rating scales;
6. Not be taking part in any other clinical trial during the period;
7. Voluntarily signed informed consent forms.

#### Exclusion criteria

Participants who experience or have one or more of the following conditions will be excluded:

1. Objective tinnitus or Ménière’s disease;
2. Tinnitus induced by otitis media, otitis interna or cerebellopontine angle tumors;
3. Postcochlear lesion;
4. Patients with severe diabetes, hypertension or cardiovascular disease, or mental disease;
5. Patients unable to read, understand and complete the forms or use the rating scales;
6. Pregnant or preparing for pregnancy.

### Randomization and blinding

One researcher screens and enrolls participants at the clinic. Patients are numbered according to registration order. After participants have completed a baseline evaluation, another researcher who is uninvolved with data collection randomly assigns them to one of four treatment groups in a 1:1:1:1 ratio using a computer-generated, blocked randomization sequence generated using SPSS 15.0 software (SPSS Inc., Chicago, IL, USA). This researcher informs the therapist of the treatment assignment.

We applied a single-blind design in which the study patients, data collocation staff, and data analysts are blinded during the study protocol. The therapists are not blinded to the treatments they deliver because treatment manipulation makes it impossible. During the intervention, therapists and data collection staff are instructed not to exchange information with each other nor communicate with the study patients.

### Interventions

**Figure 2:**
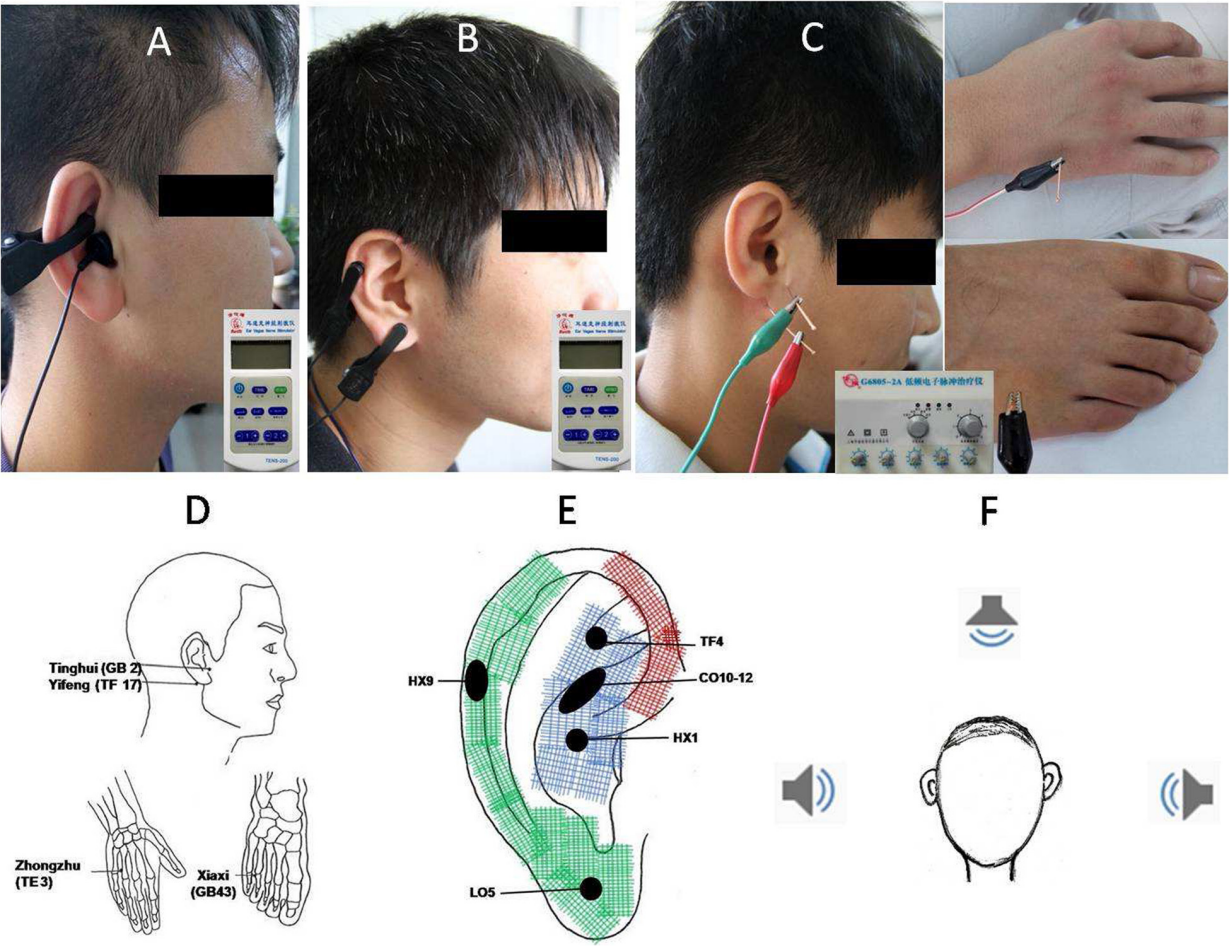
**Locations and stimulation devices. (A)** Points used in the ABVN group and the ABVN plus tone group for transcutaneous electrical nerve stimulation. **(B)** Points used in the non-ABVN plus tone group for transcutaneous electrical nerve stimulation. **(C)** Acupoints used in the electrical acupuncture group. **(D)** Locations of acupoints in the acupuncture group. local acupoints: Tinghui (GB2) and Yifeng (TF17); distal acupoints: Zhongzhu (TE3) and Xiaxi (GB43). **(E)** Innervation of the human auricle, including ABVN (blue grid), great auricular nerve (green grid) and auriculotemporal nerve (red grid) [17]; the black areas show the specific auricular acupoints. TF4, CO10-12, and HX1 are used in the ABVN group and the ABVN plus tone group; HX9 and LO5 are used in the non-ABVN plus tone group. **(F)** Ideograph of sound (tones) stimulus: each speakers is equidistant from the head of the subject.

Specific interventions of each group are as follows (Figure 2).

### ABVN group

All participants will receive transcutaneous electrical stimulation at auricular acupoints innervated by the ABVN. The points are located at the cymba conchae and the triangular fossa, where there is rich ABVN distribution [17]. Main stimulation points according to standard practice include Kidney (CO10), Yidan (CO11), Liver (CO12), and Shenmen (TF4). A transcutaneous electrical nerve stimulator (Suzhou Medical Appliance Co. Ltd., Suzhou, China) will be used in this group. Two carbon-impregnated silicone electrode tips are connected to the transcutaneous electrical nerve stimulator by metal wire; one tip contacts the skin surface points and the other acts as a terminal end in the ear. The stimulation frequency is 20 Hz, the stimulation current is 1 to 5 mA, with stimulus pulses shorter than 1 ms in duration. Each session will last 30 minutes; sessions are performed every other day for 8 weeks.

### ABVN plus tone group

The format will be exactly the same as for the ABVN group. Multiple tones will be delivered by Tinnitus Measurer software (Neonix, USA) at a comfortable listening level during the transcutaneous electrical nerve stimulation. Randomly interleaved pure tones that span the hearing range but exclude the tinnitus tone will be selected. Each session will last 30 minutes; sessions are performed every other day for 8 weeks.

### Non-ABVN plus tone group

The format of this group will be the same as for the ABVN plus tone group, except that the subjects receiving stimulation will be stimulated at auricular acupoints supplied by the great auricular nerve [17]. Each session will last 30 minutes; sessions are performed every other day for 8 weeks.

### Electrical acupuncture group

The subjects of this group will be treated with electrical acupuncture at local and distal points. Acupuncture points are selected as follows. 1. local acupoints: Yifeng (TE17), Tinghui (GB2); 2. distal acupoints: Xiaxi (GB43), Zhongzhu (TE3). Patients will be in a comfortable and relaxed position and asked to concentrate on the treatment task. After the needles are inserted into the acupoints, they will be connected to an electrical point stimulation device (G6805-2A, Shanghai Huayi Medical Instrument CO., Ltd, Shanghai, China) operating at a frequency of 20 Hz and current of 1 to 5 mA, with pulses shorter than 1 ms in duration. The intensity will be adjusted individually based on the tolerance of the patient. All parameters of the electrical stimulation inducing twitching of the muscles indicate effective stimulus. After retaining the needles for 30 min, all needles are taken out using alcohol-soaked cotton balls to avoid infection and bleeding.

The needles (Suzhou Medical Supplies Factory Co., Ltd. Suzhou, China) used in this group are 25 mm in length and 0.3 mm in diameter. Electrical acupuncture is performed by a therapist with rich clinical experience and acupuncture licenses for Chinese medicine practitioners from the Ministry of Health of China. Treatment will be conducted over a period of 8 weeks, at a frequency of every other day.

### Primary outcome measures

The primary outcome is evaluated using the Tinnitus Handicap Inventory, which evaluates 25 items grouped into a functional subscale (11 factors), an emotional subscale (9 factors), and a catastrophic subscale (5 factors). Each question of the Tinnitus Handicap Inventory can be answered by either ‘often’ (4 points), ‘sometimes’ (2 points), or ‘never’ (0 points). The total score allows the tinnitus to be categorized as: ‘slight’ (0 to 16 points), ‘mild’ (18 to 36 points), ‘moderate’ (38 to 56 points), ‘severe’ (58 to 76 points), or ‘catastrophic’ (78 to 100 points). The assessment is at baseline (before randomization), and in the 4th and 8th week, with further follow-up visits after termination of the treatment at the 12th week.

### Secondary outcome measures

A visual analogue scale, measuring the disturbance and the loudness of the tinnitus will be used. This consists of 100 mm lines with ‘0 = total absence’ and ‘100 = maximum’ of tinnitus disturbance and loudness, respectively.

The severity of both anxiety and depression is evaluated using the Hospital Anxiety and Depression Scale, in which seven items for anxiety and another seven items for depression are assessed. Each item is answered by the patients on a four point (0 to 3) response category. A score of 0 to 7 for either subscale could be regarded as being in the normal range, a score of 8 to 10 is just suggestive of the presence of the respective state and a score of 11 or higher indicates the probable presence of the mood disorder [20]. Both of these assessments will be used at baseline (before randomization), and in the 4th and 8th week, with further follow-up visits after termination of the treatment at the 12th week.

### Statistical methods

The trial aims to detect a difference between the four study groups. We present here our power analysis for the primary outcome only. This is deemed a clinically significant difference of a 20-point or greater reduction in score on the Tinnitus Handicap Inventory following treatments. Since this is a novel therapy, we used data from a previous proof-of-concept feasibility study [21], the mean decrease rate is 28% in the VNS group and 0.97% in the control group.

The following formula was used for a four-group trial [22]:

$$ {n}_1={n}_2=\frac{{\left({\mu}_{\alpha }+{\mu}_{\beta}\right)}^22P\left(1-P\right)}{{\left({P}_1-{P}_2\right)}^2} $$

Calculations are performed using 80% power and a 5% significance level (two-side). The required sample size is approximately 27 participants for each group. Assuming a 10% dropout rate, we plan to enroll a total of 120 participants with four groups of equal size (30 participants per group).

### Statistical analysis

In this trial, our primary outcome measure is the Tinnitus Handicap Inventory. We define the treatment response as a reduction in score on the Tinnitus Handicap Inventory following treatments of 20 points or more. First, baseline characteristics will be analyzed using descriptive statistics for each group. Then repeated measures analysis is performed to make comparisons among the treatment groups and the control group (ABVN plus tone versus electrical acupuncture, non-ABVN plus tone versus electrical acupuncture, and ABVN versus electrical acupuncture) at different time points (4th, 8th, and 12th week). If a significant difference is found, the next step is to make comparisons among the three treatment groups in effectiveness. Multiple comparisons will be adjusted according to the Bonferroni correction method. The Kruskal-Wallis test will be employed in the analysis of skewed distribution data. Analysis of variance will be used for numerical variables, and the *χ*^2^ test for categorical variables.

Statistical analysis will be performed by the Medical College of China Three Gorges University, No.8, University Avenue, Yichang, Hubei, China. The statistician is blinded from the allocation of groups. The SPSS 15.0 software package (SPSS Inc., Chicago, IL, USA) will be used to analyze the data.

### Participant protections and ethics

The protocol adheres to the latest revision of the Helsinki Declaration and the Chinese law of human study and has been approved by the Research Ethical Committee of Yichang Hospital of Traditional Chinese Medicine, Clinical Medical College of Traditional Chinese Medicine, China Three Gorges University. The participants are informed of the potential benefits, risks, alternatives, and responsibilities during the study before they are asked to provide consent. Potential mild adverse events, reported to be related to electro-acupuncture treatment, are pain, bleeding, tiredness, and a feeling of faintness in the acupuncture group [23], and skin redness and pressure marks in the other groups, but these mild symptoms will generally resolve spontaneously after treatment. Additionally, ABVN is thought to be involved in some peculiar somatovisceral reflexes. For instance, an ear-cough reflex (Arnold’s reflex) is estimated to be present in approximately 4% [24] of the general population, while there is rare occurrence of ear-gag reflex, ear-lacrimal reflex and auricular syncope [24,25]. So there is a rare possibility that patients in the two groups receiving ABVN stimulation might have these reflexes. Participants who show any adverse effects during the course of the therapy will receive appropriate treatment immediately. All adverse effects will be documented, and patients with persistent worsening symptoms will be withdrawn from the study.

## Discussion

We have presented the design and protocol for the randomized controlled clinical study for tinnitus patients. If it proves successful, this new treatment method could offer hope to millions of patients who are affected by tinnitus.

Although the exact pathophysiology of tinnitus still remains elusive, it is believed that an abnormal balance between inhibition and excitation causing map reorganization of central auditory circuits underlies many forms of tinnitus [26]. Moreover, signs of abnormal activity were not only revealed in the central auditory system but also in non-auditory areas, including the limbic system [27-29]. The annoyance of tinnitus is not correlated with its acoustic characteristics, but there is a significant correlation with psychological symptoms [30]. The autonomic nervous system is a major factor in the difference between simply perceiving tinnitus and being distressed by it [31]. Studies have reported that tinnitus distress correlated positively with sympathetic markers and negatively with parasympathetic markers [32,33]. Accumulating evidence illustrates that tinnitus is correlated with symptoms of distress, such as emotional stress and depression for the important role of the limbic system in the pathophysiology of tinnitus, which is based on the further understanding of auditory-limbic interactions [34-36].

Vagal nerve stimulation was developed based on this growing understanding of tinnitus. It may work by activating the nucleus of the solitary tract, which, in turn, may activate the locus coeruleus and nucleus basalis, which release neuromodulators that have significant effects on learning and memory and in plasticity regulation by modulating neurons in the cortex, hippocampus, and amygdala [26]. Engineer and colleagues [8] have indicated that cervical VNS paired with specific auditory stimuli completely eliminated the physiological and behavioral correlates of the phantom sound. They observed that pairing tones with VNS significantly acutely increased excitability and suppressed spontaneous multi-unit activity in rat auditory cortex [37]. Indeed, beneficial effects in relieving psychological symptoms have been confirmed in patients with tinnitus using invasive VNS pairing tones [21] or transcutaneous VNS paired with tones [19,38] in recent small sample pilot studies. However, another experiment also demonstrated that VNS-directed long-lasting reversal of pathological neural plasticity is driven by the repeated association of VNS with tones, and not by VNS alone [8]. German researchers [14] claimed that no clinically relevant improvement of tinnitus complaints was observed after transcutaneous VNS alone. To some extent, this may confirm the suggestion that stimulation at ABVN might prove less effective than VNS because fibers from the auricular branch do not primarily target the nucleus of the solitary tract and so only target it partly. Otherwise, as these researchers themselves suggested [14], the most probable explanation is that VNS alone is not effective in reducing tinnitus, which is supported by the work of Engineer *et al*. [8]. While sound stimulus alone, as a common sensory exposure treatment strategy, has been applied to treat tinnitus for many years [39,40], studies have indicated that it has only provided some temporary relief [41,42]. Therefore, to identify whether ABVN or the combination of ABVN and sound stimulus plays a more important role in relieving tinnitus, we designed this four-armed novel trial to determine their specific effect on tinnitus.

Acupuncture is an important part of traditional Chinese medicine. It has been accepted in China for thousands of years and is now used as an alternative and complementary medical therapy in Western countries. Meta-analysis results have indicated that acupuncture or acupuncture combined with other therapies is superior to medication alone or non-acupuncture treatments in treating subjective tinnitus [43]. However, in another systematic review, acupuncture for the treatment of tinnitus has not been demonstrated to be efficacious [44]. Whether acupuncture is effective or not in the treatment of tinnitus is a question worthy of discussion. According to the theory of traditional Chinese medicine, the shaoyang meridians of the hand and foot travel to the ear region, so acupoints of the shaoyang meridians located both in local and distant areas of the ear are used to treat tinnitus in this protocol. In addition, the specific acupoints Yifeng (TE17), Tinghui (GB2), Xiaxi (GB43), and Zhongzhu (TE3) are usually chosen in treating tinnitus.

Auricular acupuncture, as an important branch of acupuncture, has also been utilized in the treatment of diseases for years. In ancient China’s earliest medical text *Huang Di Nei Jing*, which was compiled in the 5th century BC, the correlation between the external ear and the body or viscera is described. In 1957, Dr. Paul Nogier, a physician in France, first originated the concept of an inverted fetus map on the external ear [45]. He proposed a theory that there is a somatotopic and viscerotopic representation on the auricle [46]. According to these theories, disorders from a particular part of the body or viscera can be treated by the corresponding points in the ear [46,47]. Therefore, related auricular acupoints such as Kidney (CO10), Yidan (CO11), or Liver (CO12) are usually selected to treat tinnitus, which, according to the organ-viscera theory of traditional Chinese medicine, results from insufficient Kidney qi or dampness-heat of the Liver and Gallbladder. The Shenmen point (TF4) has the effect of tranquilizing the mind [48,49]. It is often utilized to remedy mental illnesses or psychiatric disorders, such as insomnia [50], anxiety [51,52], or tinnitus [53]. However, the physiological mechanisms associated with these auricular acupoints remain unclear. In this study, it is proposed that the autonomic and the central nervous system could be modified by auricular vagal stimulation via projection from the ABVN to the nucleus of the solitary tract [54], which might work through the neuromodulator system, thus directing plasticity to treat tinnitus.

However, our study has several potential limitations. First, all the outcome measures are self-assessments instead of objective measures. To some extent, objective measures carry out relatively convincing evidence. However, the Tinnitus Handicap Inventory [55,56], visual analogue scale [56], and Hospital Anxiety and Depression Scale [57] have been much used in most of the randomized controlled trials on tinnitus and they are proved to be efficient in reflecting the subjective emotional changes related to tinnitus. Second, this trial is single-blind, therefore, the subjective factors from therapists might bias the outcome assessments. However, it is hard to blind the therapists, who are required to be familiar with treatments for the specific grouping assignment. Another limitation concerns the fact that this is a single center study. It does not lend results to great generalizability to more diverse sets of patients in more diverse regions. In addition, the sample size in this study is relatively small. A large sample, multicenter, and objective measures to assess the effectiveness of treatment should be included in a future study.

## Trial status

This trial is currently recruiting participants.

### Consent

Written informed consent was obtained from the participants for publication of this manuscript and accompanying images. A copy of the written consent is available for review by the editor-in-chief of this journal.

## Abbreviations

ABVN: Auricular branch of vagus nerve
VNS: Vagus nerve stimulation
